# Supplementary material for: Impacts of shared mobility on vehicle lifetimes and on the carbon footprint of electric vehicles
Source: Nat Commun. 2022 Oct 27;13:6400. doi: 10.1038/s41467-022-33666-2 (PMC9613654; doi:10.1038/s41467-022-33666-2)
Supplement: Supplementary file 1 — Supplementary Information [file 41467_2022_33666_MOESM1_ESM.pdf]

Impacts of shared mobility on vehicle lifetimes and the carbon footprint of electric vehicles

Johannes Morfeldt[1,*], Daniel J. A. Johansson[1]

[1] Physical Resource Theory, Department of Space, Earth and Environment, Chalmers University of Technology, Maskingränd 2, SE-412 96 Gothenburg, Sweden.

* Corresponding author. E-mail: johannes.morfeldt@chalmers.se (J. Morfeldt)

Supplementary Information

Supplementary Tables 2

Supplementary Figures 4

Supplementary Notes 12

Supplementary Note 1: Limited data on vehicle retirement of EVs confirm trends 12

Supplementary Note 2: Batteries may outlive vehicles 12

Supplementary Note 3: Statistical analysis of self-reported data on Tesla cars 13

Supplementary References 14

Supplementary Tables

Supplementary Table 1. **Criteria for filtering of the dataset.** 2

Supplementary Table 2. **Number of observations before and after filtering per engine type.** 2

Supplementary Table 3. **Number of observations before and after filtering per year of deregistration.** 2

Supplementary Table 4. **Number of observations for each stratum and the random sample size used for each stratum.** 2

Supplementary Table 5. **R-packages used for the statistical analyses.** 3

Supplementary Table 6. **Parameters estimated based on the full dataset.** 3

Supplementary Table 7. **Parameters estimated using maximum likelihood.** 3

Supplementary Table 8. **Number of entries per driving intensity class in self-reported Tesla dataset after filtering.** For Supplementary Note 3. 3

Supplementary Table 9. **Number of entries per completed cycles class in self-reported Tesla dataset after filtering.** For Supplementary Note 3. 3

Supplementary Figures

Supplementary Figure 1. **Carbon intensity of electricity generation.** 4

Supplementary Figure 2. **Distribution of annual average driving intensities.** 4

Supplementary Figure 3. **Distribution of total lifetime distance.** 4

Supplementary Figure 4. **Distribution of vehicle lifetimes.**. 5

Supplementary Figure 5. **Statistical analysis by weight class.** 5

Supplementary Figure 6**. Semi-empirical model results.** 6

Supplementary Figure 7. **Semi-empirical model results.** 7

Supplementary Figure 8. **Steady-state fleet dynamics.** 8

Supplementary Figure 9. **Sensitivity analysis – impact of lifetime-intensity model on carbon footprint.** 9

Supplementary Figure 10. **Sensitivity analysis – breakeven level of empty travel** 10

Supplementary Figure 11. **Statistical analysis of vehicle retirements for electrified vehicles.** 11

Supplementary Figure 12. **Analysis of remaining battery capacity for BEVs.** 11

Supplementary Tables

Supplementary Table 1. **Criteria for filtering of the dataset.** Note that some observations may be removed due to not fulfilling several of the criteria. Hence, the sum of observations removed will not be equal to the actual number of observations removed when considering all criteria.

Criteria	No. of observations removed	Reasoning
Age or distance travelled must not be missing, equal to zero or equal to 999999	59,159	
Time between last inspection and date of deregistration must not be longer than 14 months	360,110	This criterion aims to ensure that the vehicle has not been sitting unused for a longer time than 14 months before scrapping. The duration was chosen since vehicles older than five years need to be inspected annually and each inspection needs to happen within 14 months of the previous inspection[1]. Hence, if the duration between the last inspection is longer than 14 months, the car has most likely been sitting unused for some time.
Time between first registration of the vehicle and the model year must not be larger than two year	73,384	This criterion aims to ensure that the vehicle was not registered in Sweden late in its lifetime, indicating that it may have been driven somewhere else. This could influence the results since driving patterns may differ between countries.
Average distance travelled must not be larger than 600 km per day	102	This criterion aims to avoid vehicles with an unreasonably high mileage compared to age, probably due to errors in the dataset. The limit of 600 km is equivalent to driving 8 h at 75 km/h per day on average.
Average distance travelled must not be smaller than 1 km per day	22,959	This criterion aims to avoid vehicles that are not used regularly, such as vintage cars that are only driven a few times per year.
Mass in running order must not be larger than 3,000 kg	235	Maximum total weight for a passenger car is 3,500 kg in Sweden. The mass in running order does not cover passengers or payload. Hence, the criterion is set at 3,000 kg to account for four passengers (75 kg each) and 200 kg payload.
Engine type is gasoline or diesel without hybridization, natural gas or ethanol	2,812	Other engine types are emerging technologies (cars using electric engines in some way). The samples are limited and may be biased by issues with immature technologies or circumstances of the niche markets.

Supplementary Table 2. **Number of observations before and after filtering per engine type.**

Engine type	Before filtering	After filtering
Gasoline	794,608	393,698
Diesel	79,332	37,151
Battery electric	171	
Mild hybrid electric	2,405	
Plug-in hybrid electric	163	
Ethanol flexifuel	12,221	9,590
Natural gas / Biogas	3,047	1,956
Others	73	

Supplementary Table 3. **Number of observations before and after filtering per year of deregistration.**

	Before filtering	After filtering
2014	173,611	85,975
2015	176,175	87,015
2016	173,291	86,108
2017	179,047	89,449
2018	189,896	93,848
Total	892,020	442,395

Supplementary Table 4. **Number of observations for each stratum and the random sample size used for each stratum.**

Average annual driving intensity class	No. observations	Random sample size
0-10,000 km/year	102,873	200
10,001-20,000 km/year	283,467	200
20,001-30,000 km/year	48,187	200
30,001-40,000 km/year	5,168	200
40,001-50,000 km/year	1,102	200
50,001-60,000 km/year	478	200
60,001-70,000 km/year	311	200
70,001-80,000 km/year	257	200
80,001-90,000 km/year	203	200

90,001-100,000 km/year	145	145

Supplementary Table 5. **R-packages used for the statistical analyses.**

Section of methodology	Purpose	Function (R-package)
Swedish vehicle retirement statistics.	Stratified random sampling	stratified (splitstackshape v.1.4.8)
	Fit distributions to datasets	fitdist (fitdistrplus v.1.1-3)
Semi-empirical lifetime-intensity model.	Optimizer for maximum likelihood estimation.	optimx (optimx v.2020-4.2)
Self-reported data on remaining battery capacity (Supplementary Note 3).	Extrapolation of data.	stat_smooth (ggplot2 v.3.3.3)<br>lm (stats v.4.1.2)
	Significance in difference of the mean between strata.	t.test (stats v.4.1.2)

Supplementary Table 6. **Parameters estimated based on the full dataset.**

	Normal distribution	Weibull distribution
D_0	14,158	14,158
τ_0	16.278	18.046
α	0.30397	0.19497

Supplementary Table 7. **Parameters estimated using maximum likelihood.**

	Normal distribution	Weibull distribution
Elasticity model		
ε	-0.66894	-0.65094
β	0.52038	0.51109
Logistic model		
L_0	28.686	29.572
L	23.098	22.755

Supplementary Table 8. **Number of entries per driving intensity class in self-reported Tesla dataset after filtering.** For Supplementary Note 3.

Average annual driving intensity class	No. of entries
0-30,000 km/year	441
30,001+ km/year	567
(of which 60,000+ km/year)	55
Total	1,008

Supplementary Table 9. **Number of entries per completed cycles class in self-reported Tesla dataset after filtering.** For Supplementary Note 3.

Completed cycles class	No. of entries
0-85 cycles/year	515
86+ cycles/year	493
Total	1,008

Supplementary Figures

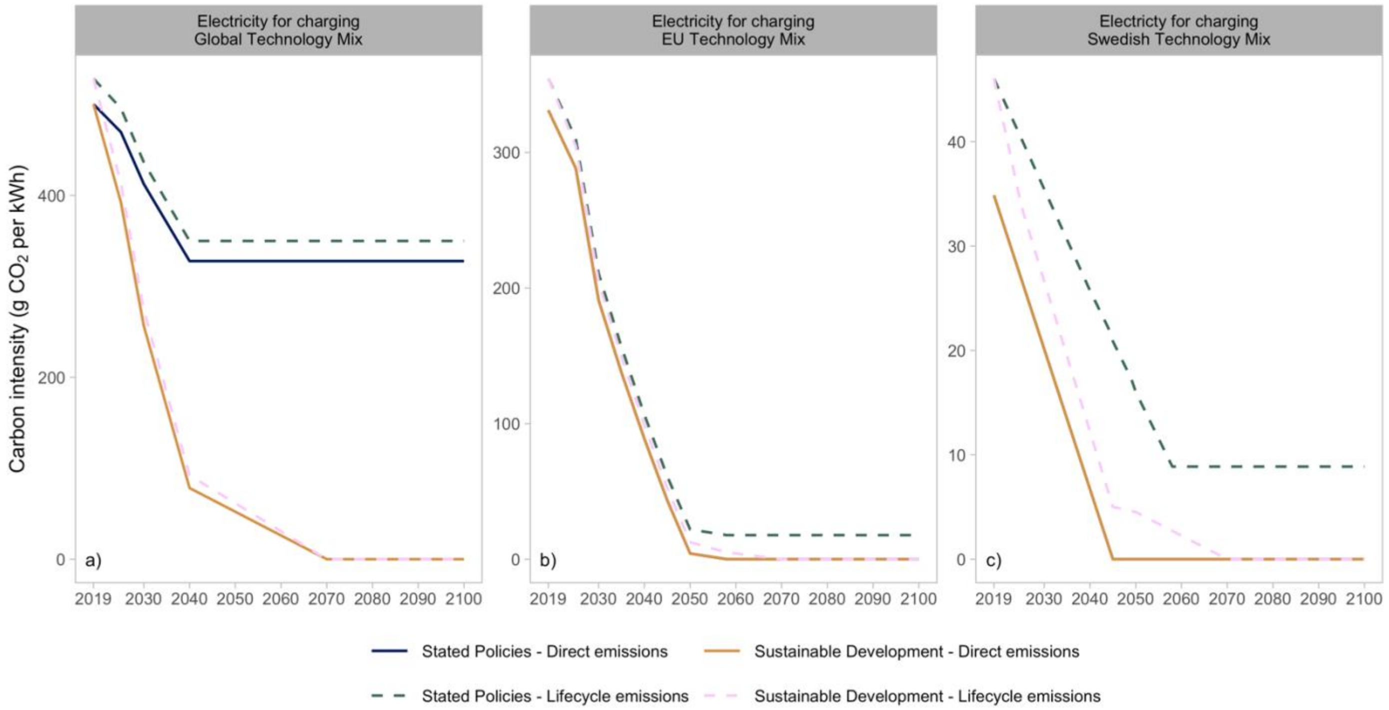

Supplementary Figure 1. **Carbon intensity of electricity generation.** **a** Global average technology mix. **b** European Union average technology mix. **c** Swedish average technology mix. Direct emissions (solid) and lifecycle emissions (dashed) are given for the two decarbonization pathways (colors). Note that direct emissions are equal between the two climate change mitigation scenarios in the cases of European and Swedish electricity mix.

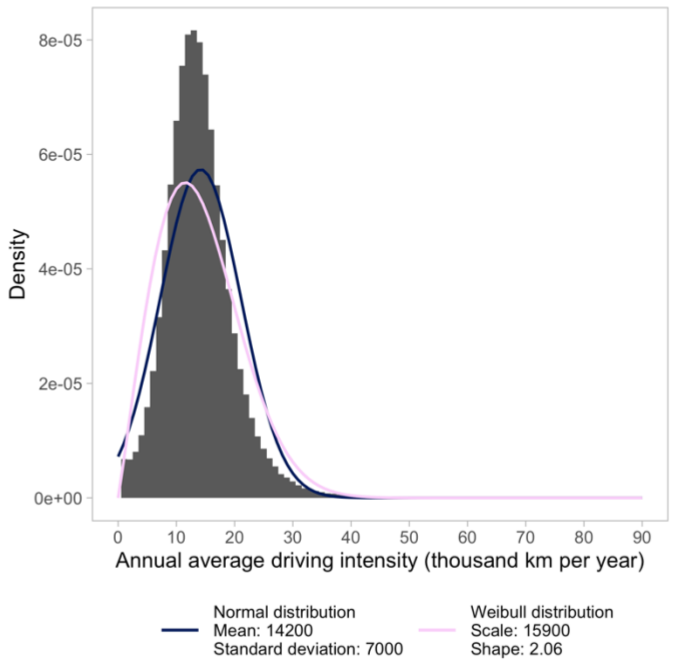

Supplementary Figure 2. **Distribution of annual average driving intensities.** Results for Swedish passenger cars retired during 2014-2018 (histogram) and fitted normal (blue line) and Weibull distributions (pink line).

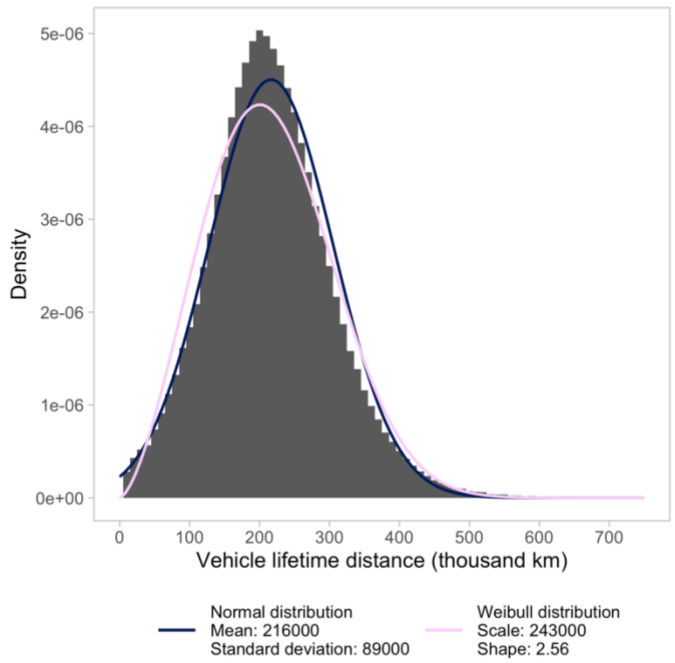

Supplementary Figure 3. **Distribution of total lifetime distance.** Results for Swedish passenger cars retired during 2014-2018 (histogram) and fitted normal (blue line) and Weibull distributions (pink line).

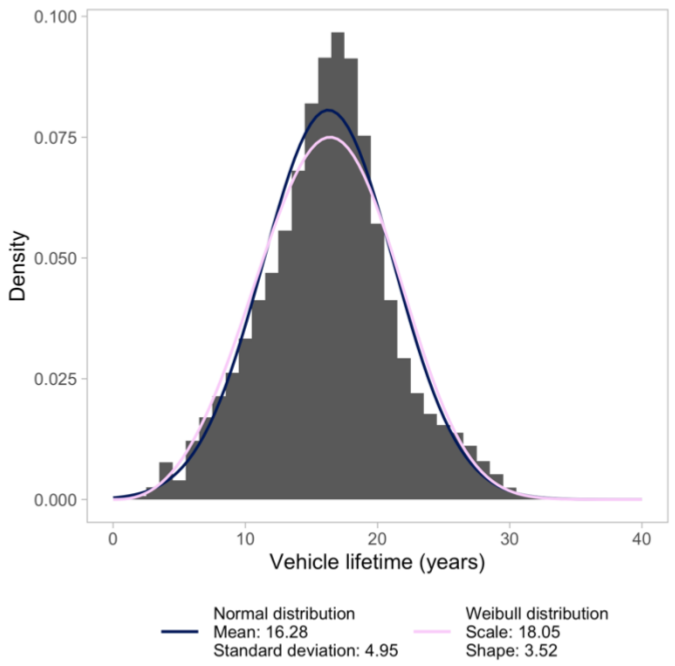

Supplementary Figure 4. **Distribution of vehicle lifetimes.** Results for Swedish passenger cars retired during 2014-2018 (histogram) and fitted normal (blue line) and Weibull distributions (pink line).

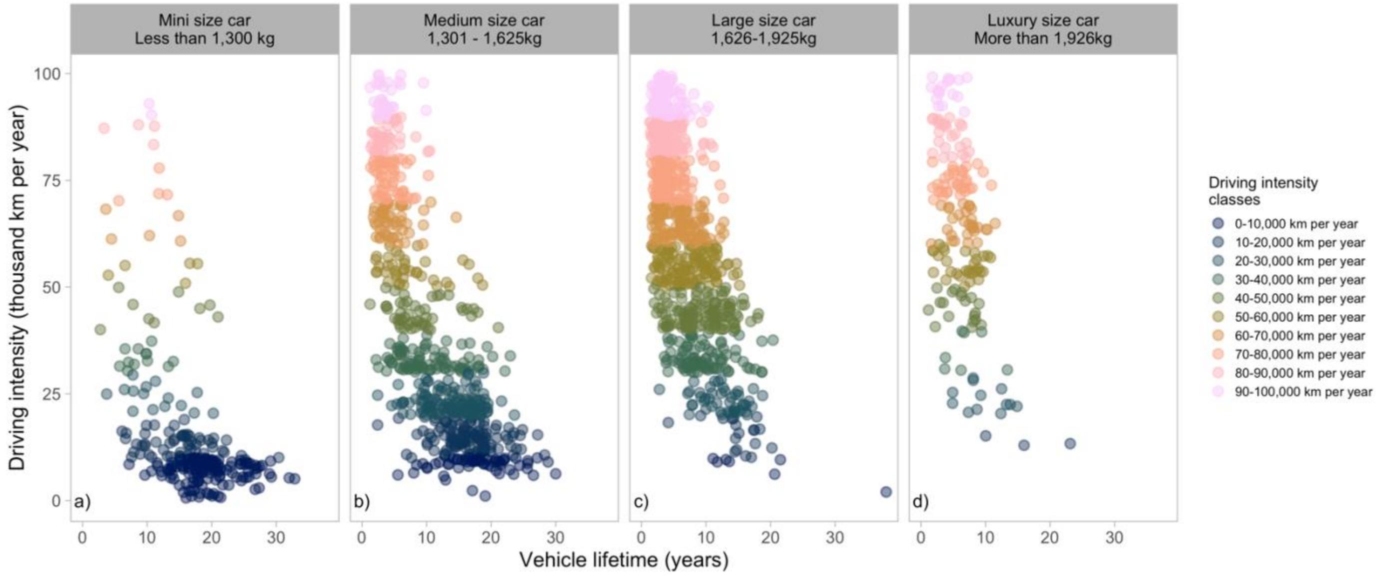

Supplementary Figure 5. **Statistical analysis by weight class. a** Mini size cars – weighing less than 1,300kg. **b** Medium size cars – weighing 1,301-1,625 kg. **c** Large size cars – weighing 1,626-1,925kg. **d** Luxury size cars – weighing more than 1,926kg, based on the classification used by Ellingsen et al.[2]. Results on vehicle lifetime and average driving intensity for stratified samples based on average driving intensity class of Swedish ICEVs retired between 2014-2018. The color indicates the driving intensity class of the data point.

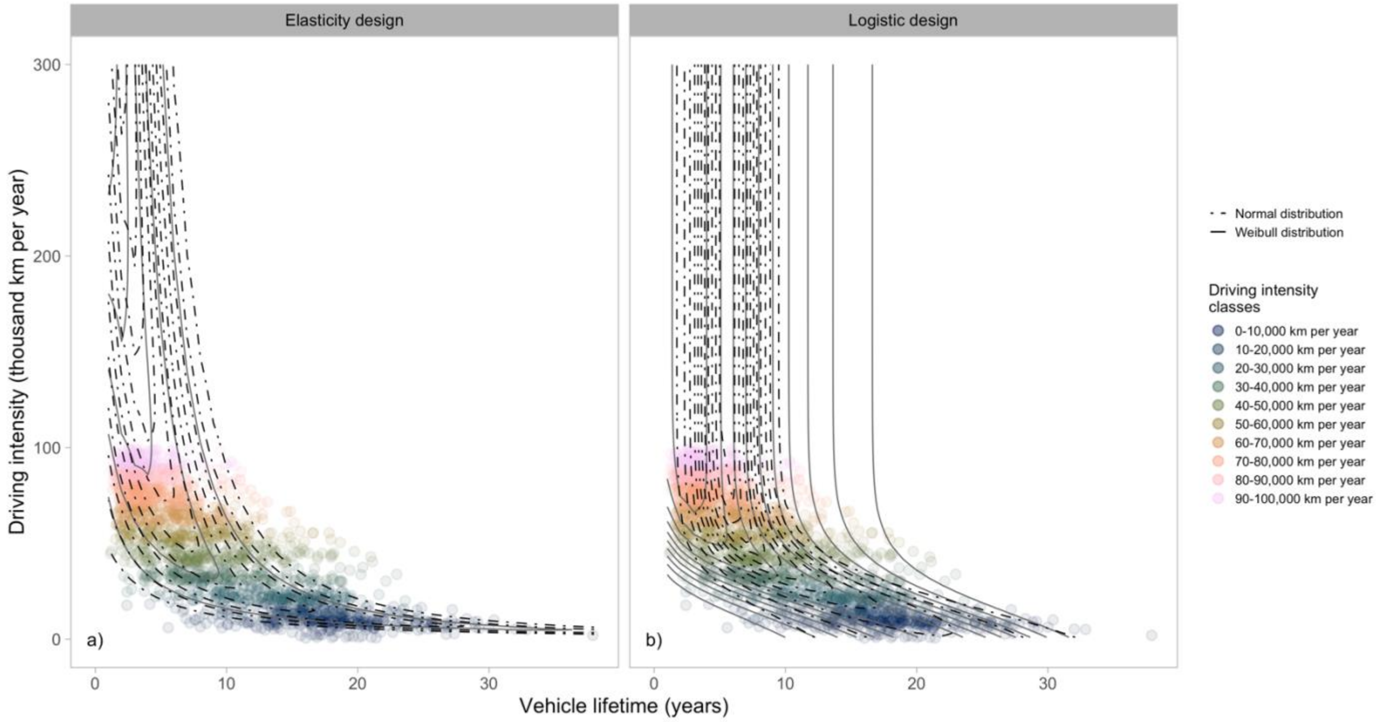

Supplementary Figure 6**. Semi-empirical model results. a** Results for the elasticity design. **b** Results for the logistic design. The contours show probability density levels and are provided for both normal (dot-dashed line) and Weibull distributions (solid line). Stratified samples of Swedish vehicle retirement statistics for 2014-2018 are provided in the background for comparison. The color indicates the driving intensity class of the data point.

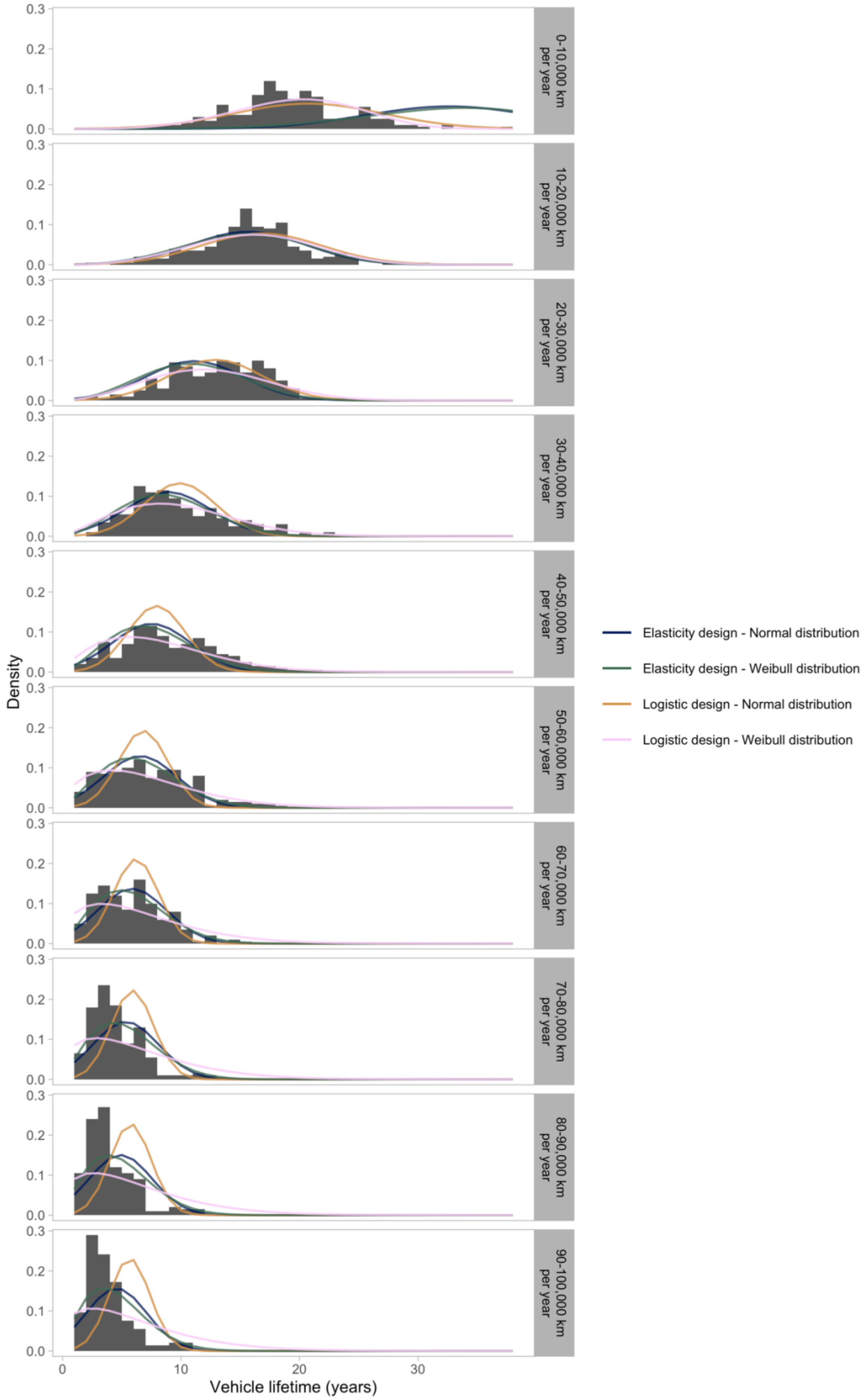

Supplementary Figure 7. **Semi-empirical model results.** Results for each respective driving intensity class (vertical panels) and modelling approach (blue: elasticity design – normal distribution, green: elasticity design – Weibull distribution, orange: logisitic design – normal distribution, pink: logistic design – Weibull distribution), compared to the distribution of the stratified sample of the same class.

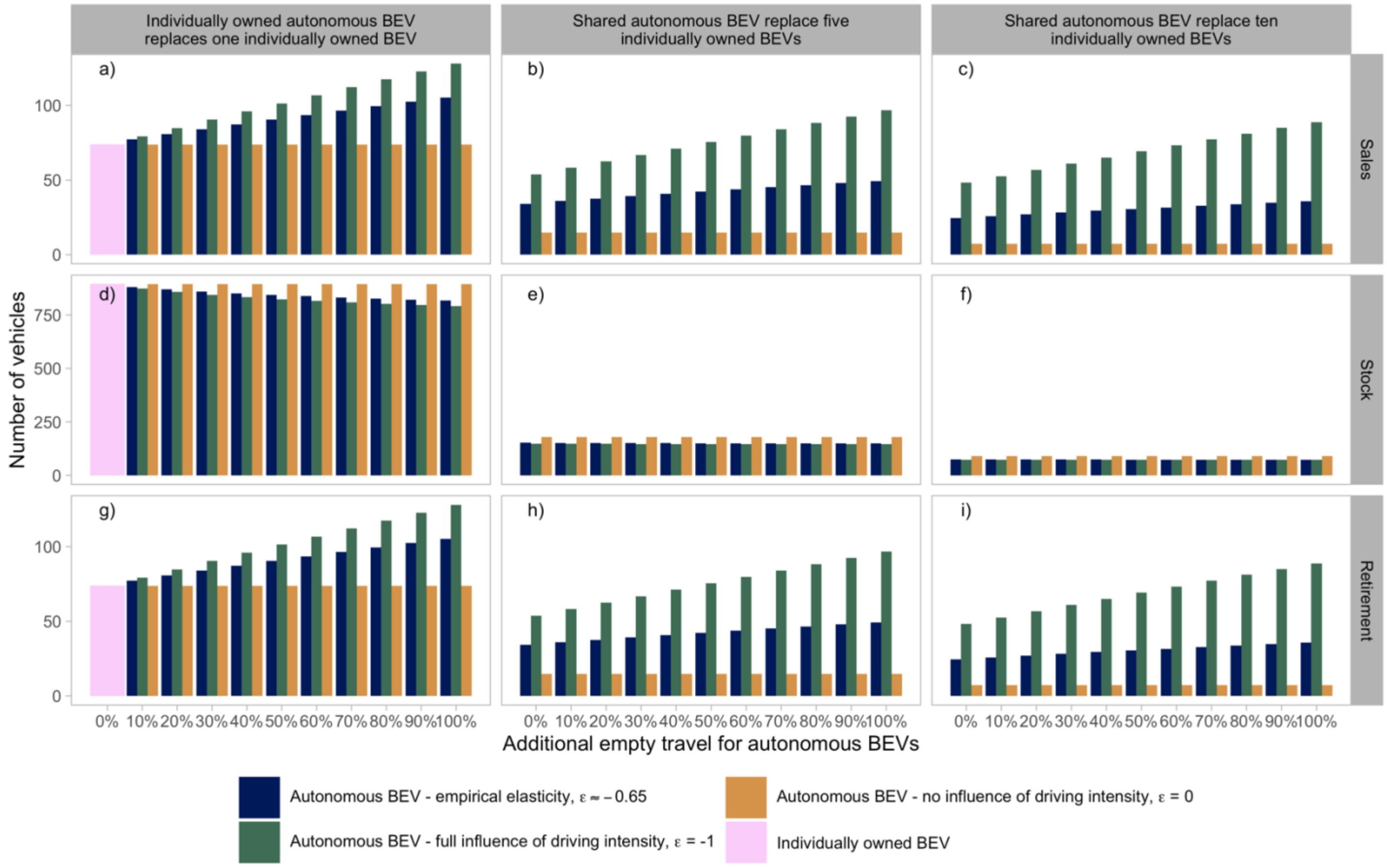

Supplementary Figure 8. **Steady-state fleet dynamics.** **a**, **b**, **c** Number of sold cars. **d**, **e**, **f** Current stock of cars. **g**, **h**, **i** Number of retired cars. Results show impact of lifetime-intensity elasticity (color) for individually owned autonomous BEVs that replace one individually owned BEV (**a**, **d**, **g**) and shared autonomous BEVs replacing five (**b**, **e**, **h**) or ten (**c**, **f**, **i**) individually owned. Results depend on the elasticity of the semi-empirical lifetime-intensity model (blue: shared autonomous BEV – empirical elasticity, $\varepsilon \approx -0.65$, green: shared autonomous BEV – full influence of driving intensity, $\varepsilon = -1$, orange: shared autonomous BEV – no influence of driving intensity, $\varepsilon = 0$, and pink: individually owned BEV – current driving intensity).

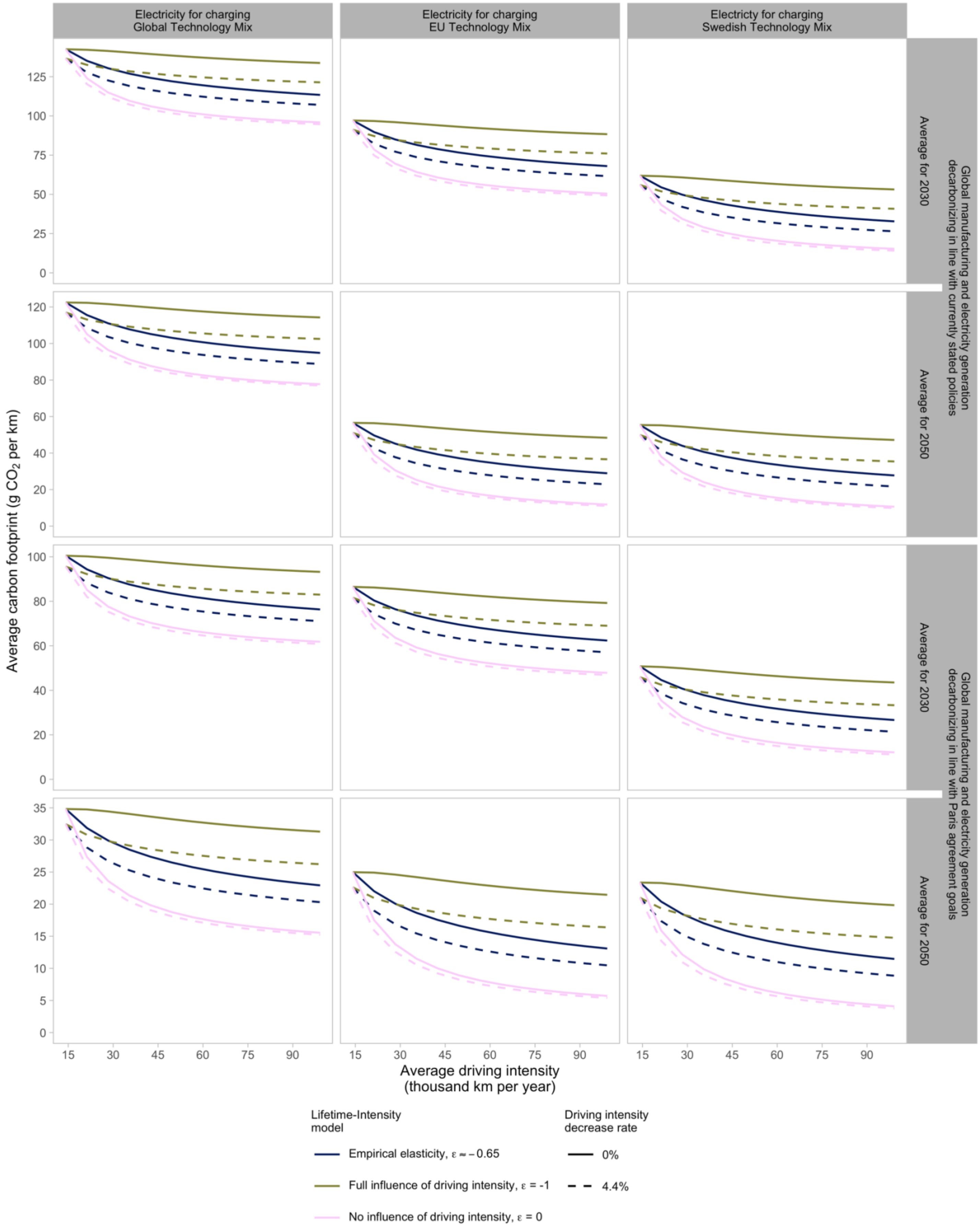

Supplementary Figure 9. **Sensitivity analysis – impact of lifetime-intensity model on carbon footprint.** Results show the impact on total carbon footprint for 2030 and 2050, depending on driving intensity decrease rate (solid: 0% and dashed: 4.4%), elasticity of the semi-empirical lifetime-intensity model (blue: empirical elasticity, ε ≈ -0.65, green: full influence of driving intensity, ε = -1, and pink: no influence of driving intensity, ε = 0), grid used for charging and decarbonization scenario for global manufacturing and electricity generation.

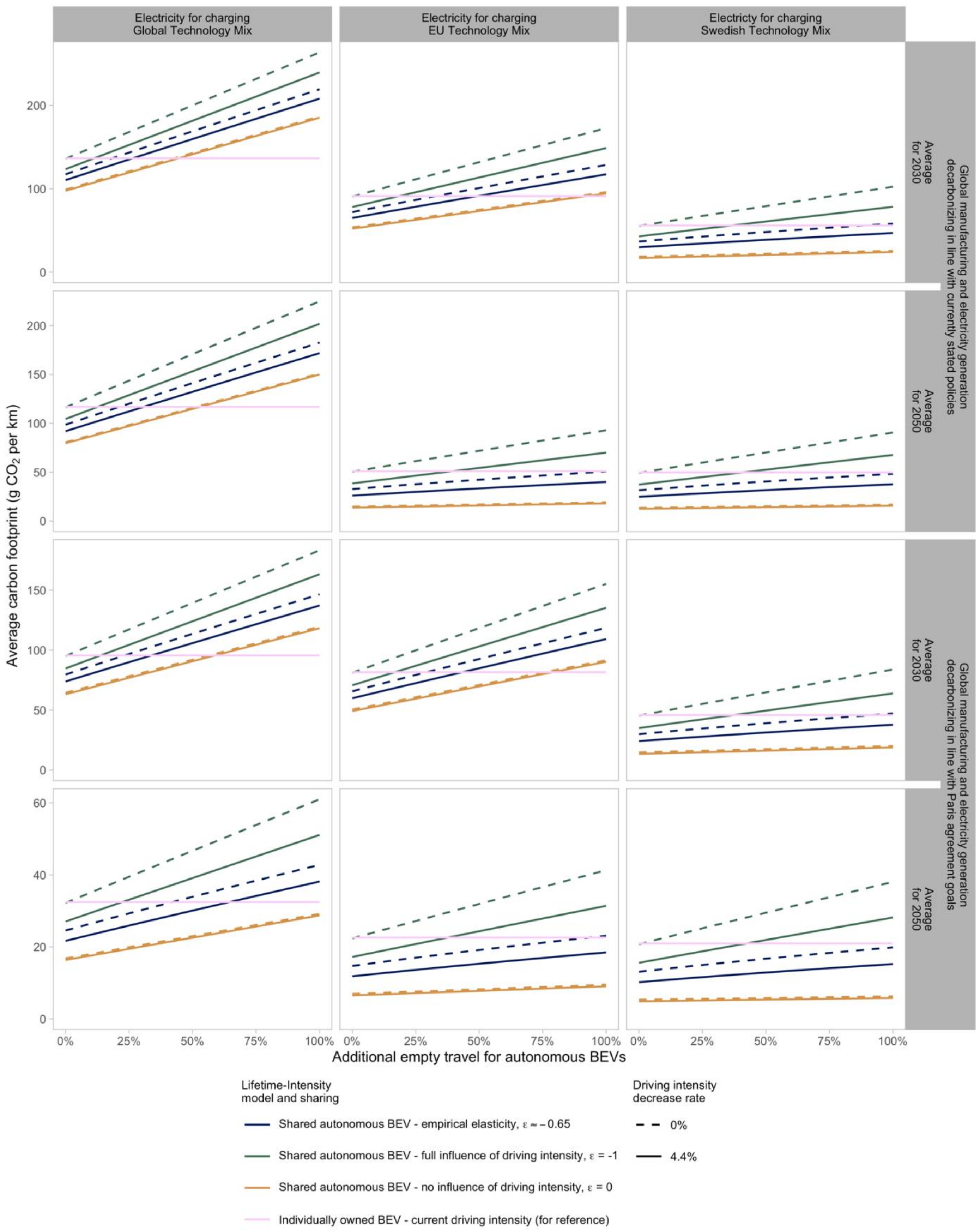

Supplementary Figure 10. **Sensitivity analysis – breakeven level of empty travel**. Results show the breakeven level for 2030 and 2050, depending on driving intensity decrease rate (solid: 0% and dashed: 4.4%), elasticity of the semi-empirical lifetime-intensity model (blue: shared autonomous BEV – empirical elasticity, ε ≈ -0.65, green: shared autonomous BEV – full influence of driving intensity, ε = -1, orange: shared autonomous BEV – no influence of driving intensity, ε = 0, and pink: individually owned BEV – current driving intensity), grid used for charging and decarbonization scenario for global manufacturing and electricity generation.

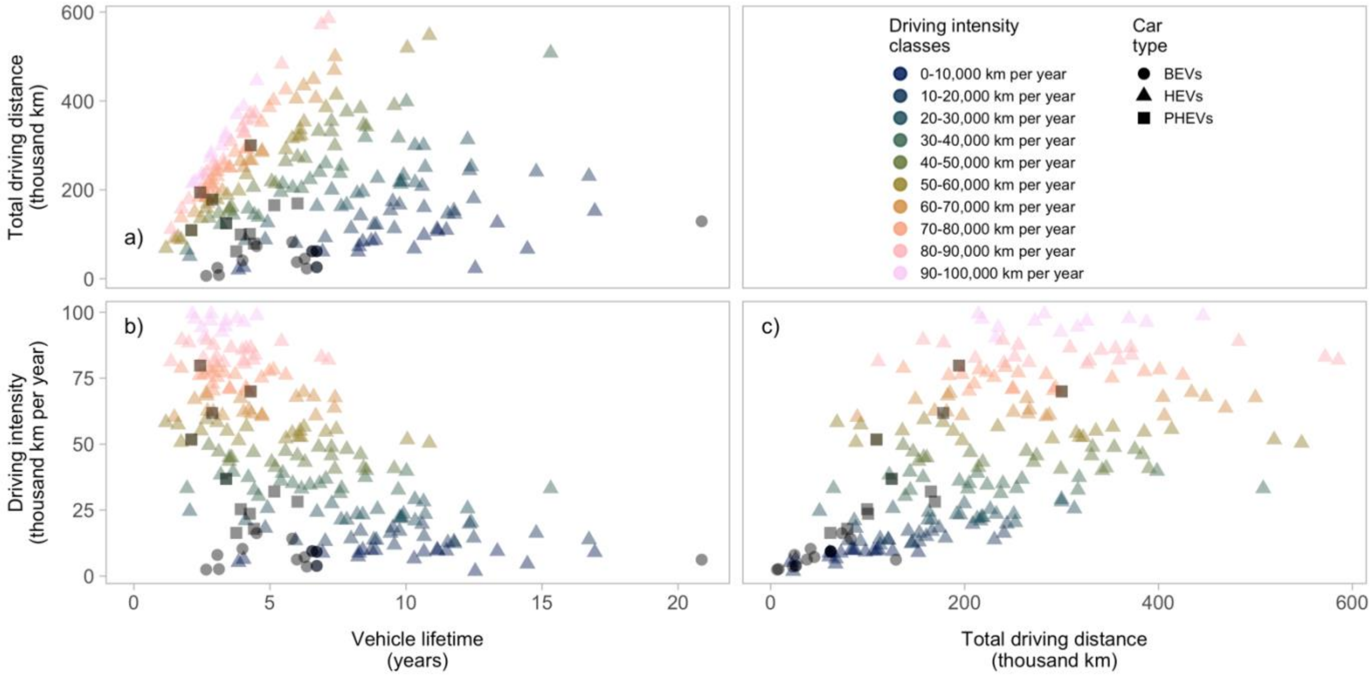

Supplementary Figure 11. **Statistical analysis of vehicle retirements for electrified vehicles. a** Vehicle lifetime and total driving distance. **b** Vehicle lifetime and average driving intensity. **c** Total driving distance and average driving intensity. Results are shown for stratified samples based on average driving intensity classes (points)of Swedish cars with batteries retired between 2014-2018. Shapes indicate the car type with a bias towards HEVs in the stratified samples (circle: BEVs, triangle: HEVs and squares: PHEVs). All BEVs and PHEVs in the filtered dataset are displayed in black. Other colors indicate the driving intensity class of the data point.

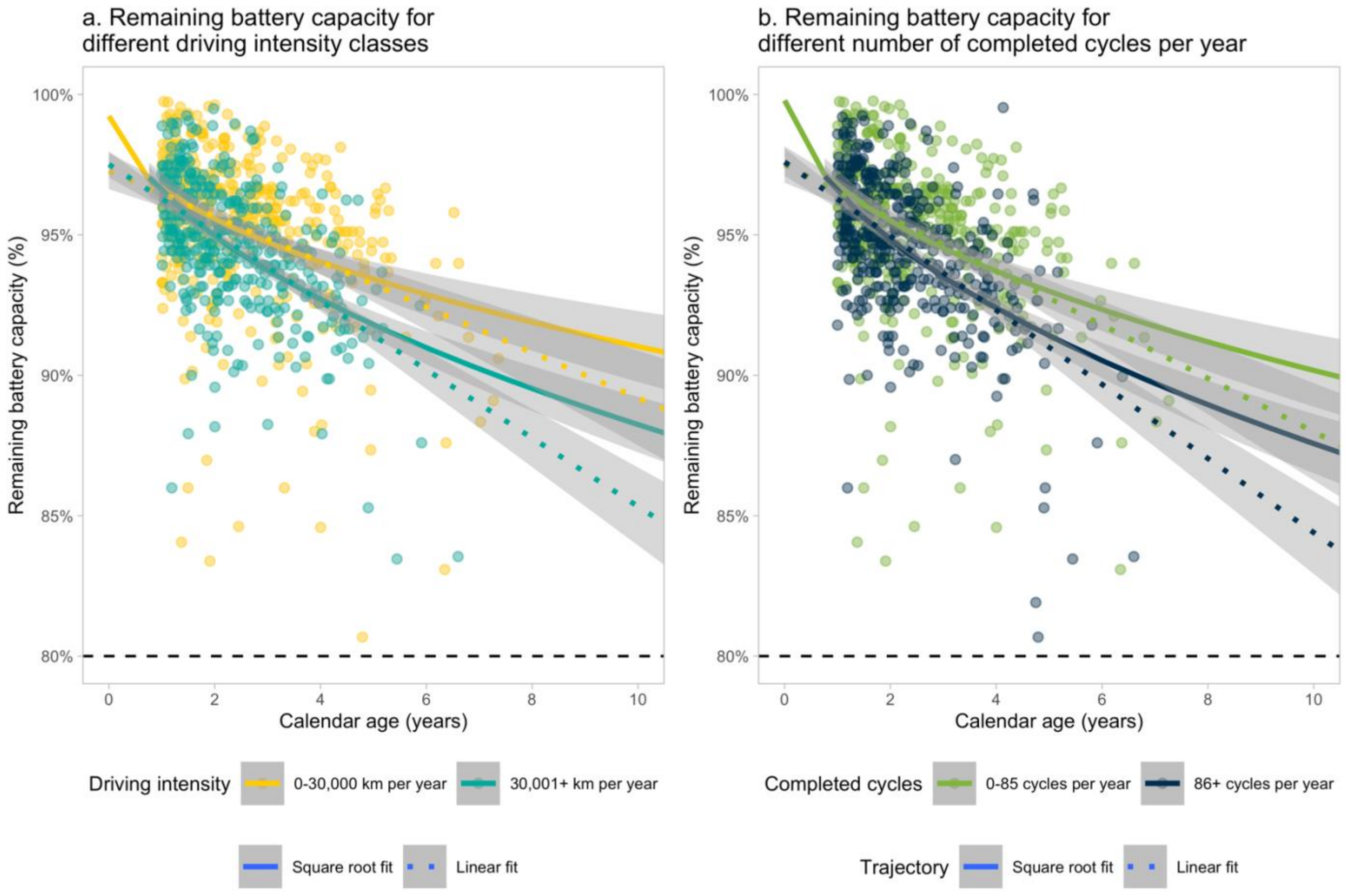

Supplementary Figure 12. **Analysis of remaining battery capacity for BEVs. a** Stratification based on driving intensity. **b** Stratification based on number of completed cycles. Results based on self-reported data from Tesla users, where line types indicate type of fit to the data (solid: square root fit and dotted: linear fit) and the colors of the dots indicate the driving intensity class (a) and completed cycle class (b).

Supplementary Notes

Supplementary Note 1: Limited data on vehicle retirement of EVs confirm trends

Although the retirement data for electric vehicles are relatively sparse, the statistics indicate a similar relationship as the one estimated for ICEVs. The results of the stratification of retirement data on cars with batteries (used for propelling the vehicle) reveal close to linear correlations between vehicle lifetime and total driving distance for individual driving intensity classes that becomes steeper with each higher class, see Supplementary Figure 11a. This suggests that the retirement age of a vehicle becomes shorter with increasing average driving intensity. Considering how the total driving distance varies with average driving intensity, see Supplementary Figure 11c, the total driving distance plays a less important role in the decision to retire a vehicle as the average annual driving intensity increases. Hence, we focus the following analysis on empirically describing the relationship between driving intensity and lifetime for electrified vehicles. The average vehicle lifetime decreases with each higher driving intensity class, from 8 years for average driving intensities of 0-10,000 km per year to 3.1 years for average driving intensities of 90,001-100,000 km per year, see Supplementary Figure 11b. Note that the average lifetime for low driving intensity classes is considerably lower for electric vehicles than for ICEVs. One key reason for this is that very few electric vehicles have been around for more than a decade, so statistical analysis precludes a relevant estimation of a relationship between driving intensity and vehicle lifetime in cases where the driving intensity is low. However, it is still clear from Supplementary Figure 11 that the vehicle lifetime tends to be shorter when driving intensity is high. Further, the standard deviation of the distributions also indicates that the range of probable lifetimes becomes narrower with increasing annual driving intensity (although the standard deviation increases in relative terms). The standard deviation decreases from 2.8 years for driving intensities of 0-10,000 km per year to 0.7 years for driving intensities of 90,001-100,000 km per year (assuming Normal-distributed data).

However, the dataset on cars with batteries is biased towards HEVs, as shown by the shapes in Supplementary Figure 11. Hence, since HEVs are primarily propelled by an ICE one can assume that the retirement decision of HEVs would be governed by similar factors as ICEVs. Supplementary Figure 11 also display the results for all data points in the filtered dataset for BEVs and PHEVs. Although EVs have not been around for enough years to draw any firm conclusions and the data points are too few for any meaningful statistical analysis , the data could follow similar patterns to ICEVs. This should be confirmed with new data as it becomes available when BEVs and PHEVs have taken a more significant share of the fleet.

Supplementary Note 2: Batteries may outlive vehicles

Battery degradation occurs over time and may be amplified by extreme ambient temperatures, storing it at high states of charge, high cycling rates (i.e., the rate of completed full charging and discharging cycles), bad thermal management and high depth of discharge (i.e., almost fully discharging the battery before connecting to a charger)[3–5]. Retirement standards are used to determine when a battery should be taken out of use, which currently, in many scientific papers, is set to when the actual battery capacity reaches 80% of the original capacity[3] or when power output is lower than the power demand. Meanwhile, the battery capacity threshold is set to 70% of the original capacity in several warranties from car manufacturers [6–8]. While the battery may very well provide sufficient power and range for the driver's needs below a battery capacity of 70-80%, safety related concerns may also limit how long the battery can be used with only partial battery capacity remaining[9].

Mathematical models for assessing battery degradation are typically using physical and chemical aspects of the battery cells and are empirically calibrated and verified based on data from laboratory experiments [3]. For example, assumptions must be made on how the temperature of the battery cells depends on the battery management system and on ambient temperature, or on how and when the driver choses to

charge the vehicle. Two recent studies have concluded that the battery capacity should not decrease below 80% regardless of charging strategy for a car that is driven 12,000 km per year[3,10].

Statistics on battery lifetimes from real-world driving are scarce due to the recent large-scale introduction of electric vehicles in the passenger car market. The number of electric vehicles on the roads in 2010 were in the thousands while it reached over 10 million by 2020. Since then there has also been a shift towards the nickel manganese cobalt oxide (NMC) battery chemistry[11,12]. If enough retirement statistics for electric vehicles were available to make thorough statistical analyses, most vehicles would only be less than 10 years old. Also, future battery chemistries may differ from the ones used today with different characteristics[13]. Hence, it would be hard to draw any distinct conclusions that could be expected to be valid also for future electric vehicles.

Supplementary Note 3: Statistical analysis of self-reported data on Tesla cars

Nevertheless, one statistics source that is available is self-reported data on the remaining battery capacity of Tesla cars over ca. 10 years of driving[14]. As per 23 January 2021, the dataset includes 1,649 entries for cars manufactured between 2012 and 2020. Each entry includes information on the Tesla model, manufacturing date, total driving distance, reporting date, frequency of supercharging and remaining battery capacity (as reported by the car computer system). Entries without manufacturing date, with age estimated to less than one year or with reported remaining battery capacity above 100% are excluded, leaving 1,008 entries in the filtered dataset.

The data was stratified into two classes either based on average annual driving intensity, 0-30,000 km/year, 30,001+ km/year, see Supplementary Table 8, or on completed number of charging cycles, 0-85 cycles/year and 86+ cycles/year, see Supplementary Table 9. The number of charging cycles are estimated based on each observations cumulative driving distance, the average specific energy use of 220 Wh per km, and each models battery capacity. Random samples containing 400 observations of each class are used in the following analysis. The data is extrapolated by fitting a linear function or a square root function to the data, see Supplementary Table 5 for details on the statistical package used. Note that Tesla cars and their battery management systems have been further developed since this database was founded and that the results therefore should be interpreted with great care.

The stratification of the dataset based on average annual driving intensity shows that more intensive driving habits slightly affects the remaining battery capacity (mean: 95.1% to 94.4%, considered statistically significant based on a Welch Two Sample t-test, and median: 95.7% to 94.7%). It is also interesting to note that the difference is even higher for cars older than 5 years (mean: 92.2% to 85.9%), although that sample only covers 28 cars. Linear and square root extrapolations[5,15,16] of the datasets illustrate that the cars could reach a remaining battery capacity below 80% by a 15-year lifetime for high annual driving intensity and a 20-year lifetime for low annual driving intensity (linear extrapolation) or a lifetime that exceeds 25 years (square root extrapolation), see Supplementary Figure 12a. Neither of the extrapolations can be seen as predictions and are provided only for illustrative purposes given that several additional aspects likely influence battery degradation that are not taken into account here. Also, using a 70% battery capacity limit would have increased the estimated battery lifetime.

The stratification of the dataset based on completed cycles shows also shows similar tendencies that more intensive driving habits in terms of completed charging cycles per year slightly affects the remaining battery capacity (mean: 95.3% to 94.5%, considered statistically significant based on a Welch Two Sample t-test, and median: 95.7% to 94.7%). The linear extrapolations of the datasets show similar results as for stratification on driving intensity (a 15-year lifetime for high number of cycles per year and a 20-year lifetime for low number of cycles per year, when assuming an 80% battery capacity limit), see Supplementary Figure 12b. However, the square root extrapolation shows slightly shorter lifetime for the high cycles per year stratum as compared with the high driving intensity stratum.

The self-reported data for Tesla and results using battery degradation models of current battery chemistries, which have been verified using real-world test vehicles[3], can provide some insight to the expected lifetime of current batteries. However, the question is how representative current estimates are for future electric vehicles given the on-going research and development of batteries. One example is the potential effects of a recent study[13] on a thermally modulated iron phosphate battery that could enable faster charging times, less influence of ambient temperature on battery capacity and output power. Such a battery is expected to have a lifetime of 51 years, covering over 900,000 km lifetime distance before reaching a capacity loss of 20%. Although promising, this technology is still in the design stage and far from dominating the market. However, technologies overcoming such barriers could very well be representing a large share of the market in the future, when carsharing is introduced at large scale.

Supplementary References

1. Swedish Transport Agency. Kontrollbesiktning av personbil och lastbil som inte överstiger 3500 kg i totalvikt (Check-up of passenger cars and other light duty vehicles below 3500 kg of curb weight). https://transportstyrelsen.se/sv/vagtrafik/Fordon/fordonsbesiktning/besiktningsregler/personbil-och-lastbil-som-inte-overstiger-3500-kg-i-totalvikt/ (2021).
2. Ellingsen, L. A.-W., Singh, B. & Strømman, A. H. The size and range effect: lifecycle greenhouse gas emissions of electric vehicles. *Environmental Research Letters* **11**, 054010 (2016).
3. De Gennaro, M. *et al.* A case study to predict the capacity fade of the battery of electrified vehicles in real-world use conditions. *Case Studies on Transport Policy* **8**, 517–534 (2020).
4. Lander, L. *et al.* Cost and carbon footprint reduction of electric vehicle lithium-ion batteries through efficient thermal management. *Applied Energy* **289**, 116737 (2021).
5. Yang, F., Xie, Y., Deng, Y. & Yuan, C. Predictive modeling of battery degradation and greenhouse gas emissions from U.S. state-level electric vehicle operation. *Nature Communications* **9**, 2429 (2018).
6. Tesla. Vehicle Warranty. https://www.tesla.com/support/vehicle-warranty (2022).
7. Kia. Warranty terms and exclusions. 1–8 https://www.kia.com/uk/owners/insurance-and-warranty/7-year-warranty/terms-and-exclusions/ (2022).
8. Volkswagen. High-voltage battery: Warranty and maintenance. 1–11 https://www.volkswagen.co.uk/en/electric-and-hybrid/should-you-go-electric/servicing/battery-maintenance-and-waranty.html (2022).
9. Lai, X. *et al.* Sorting, regrouping, and echelon utilization of the large-scale retired lithium batteries: A critical review. *Renewable and Sustainable Energy Reviews* **146**, 111162 (2021).
10. Paffumi, E. & Martini, G. Real-world mobility and environmental data for the assessment of in-vehicle battery capacity fade. *World Electric Vehicle Journal* **12**, (2021).
11. International Energy Agency (IEA). *Global EV Outlook 2020 - Entering the decade of electric drive?* (2020) doi:10.1787/9789264302365-en.
12. International Energy Agency (IEA). *Global EV Outlook 2021 - Accelerating ambitions despite the pandemic*. https://www.iea.org/reports/global-ev-outlook-2021 (2021).
13. Yang, X. G., Liu, T. & Wang, C. Y. Thermally modulated lithium iron phosphate batteries for mass-market electric vehicles. *Nature Energy* **6**, (2021).
14. Teslike. Tesla Battery Survey. https://docs.google.com/forms/d/e/1FAIpQLSfrteuONm9qylEh0lgLTu6FzP2qR8-3mHTt6e53849ombZ03w/viewform (2021).
15. Schimpe, M. *et al.* Comprehensive Modeling of Temperature-Dependent Degradation Mechanisms in Lithium Iron Phosphate Batteries. *Journal of The Electrochemical Society* **165**, A181–A193 (2018).
16. Guo, J., Li, Z. & Pecht, M. A Bayesian approach for Li-Ion battery capacity fade modeling and cycles to failure prognostics. *Journal of Power Sources* **281**, 173–184 (2015).
