# Supplementary material for: Impacts of shared mobility on vehicle lifetimes and on the carbon footprint of electric vehicles
Source: Nat Commun. 2022 Oct 27;13:6400. doi: 10.1038/s41467-022-33666-2 (PMC9613654; doi:10.1038/s41467-022-33666-2)
Supplement: Supplementary file 3 — Reporting Summary [file 41467_2022_33666_MOESM3_ESM.pdf]

nature research

Corresponding author(s): Dr. Johannes Morfeldt

Last updated by author(s): Aug 24, 2022

Reporting Summary

Nature Research wishes to improve the reproducibility of the work that we publish. This form provides structure for consistency and transparency in reporting. For further information on Nature Research policies, see our Editorial Policies and the Editorial Policy Checklist.

Statistics

For all statistical analyses, confirm that the following items are present in the figure legend, table legend, main text, or Methods section.

n/a	Confirmed	
☐	☒	The exact sample size (*n*) for each experimental group/condition, given as a discrete number and unit of measurement
☐	☒	A statement on whether measurements were taken from distinct samples or whether the same sample was measured repeatedly
☐	☒	The statistical test(s) used AND whether they are one- or two-sided<br>*Only common tests should be described solely by name; describe more complex techniques in the Methods section.*
☐	☒	A description of all covariates tested
☐	☒	A description of any assumptions or corrections, such as tests of normality and adjustment for multiple comparisons
☐	☒	A full description of the statistical parameters including central tendency (e.g. means) or other basic estimates (e.g. regression coefficient) AND variation (e.g. standard deviation) or associated estimates of uncertainty (e.g. confidence intervals)
☒	☐	For null hypothesis testing, the test statistic (e.g. F, t, r) with confidence intervals, effect sizes, degrees of freedom and P value noted<br>*Give P values as exact values whenever suitable.*
☒	☐	For Bayesian analysis, information on the choice of priors and Markov chain Monte Carlo settings
☒	☐	For hierarchical and complex designs, identification of the appropriate level for tests and full reporting of outcomes
☒	☐	Estimates of effect sizes (e.g. Cohen's *d*, Pearson's *r*), indicating how they were calculated

Our web collection on statistics for biologists contains articles on many of the points above.

Software and code

Policy information about availability of computer code

Data collection	Statistics on vehicle retirement from the Swedish registry for road transport vehicles (product "Fordon på väg"), regulated by Swedish law, are provided by the government agency Transport Analysis. Life cycle assessment data are based on GREET 2 - Version 2019 that was adapted for scenario analysis by Morfeldt et al. 2021. Further details are provided in Methods and the Supplementary Information.
Data analysis	The data is analyzed with R (version 4.1.2).

For manuscripts utilizing custom algorithms or software that are central to the research but not yet described in published literature, software must be made available to editors and reviewers. We strongly encourage code deposition in a community repository (e.g. GitHub). See the Nature Research guidelines for submitting code & software for further information.

Data

Policy information about availability of data

All manuscripts must include a data availability statement. This statement should provide the following information, where applicable:

- Accession codes, unique identifiers, or web links for publicly available datasets
- A list of figures that have associated raw data
- A description of any restrictions on data availability

Data for all figures and additional data used in the analyses are available from the corresponding author upon request. Note that the detailed data on vehicle retirement are treated as confidential since data that could be traced back to individuals or companies are protection under the Swedish Public Access to Information and Secrecy Act (SFS 2009:400). Hence, requests for access to these detailed data should be made directed to the Swedish governmental agency Transport Analysis (https://www.trafa.se/vagtrafik/fordon/ - dataset “Fordon på väg”).

Field-specific reporting

Please select the one below that is the best fit for your research. If you are not sure, read the appropriate sections before making your selection.

☐ Life sciences ☒ Behavioural & social sciences ☐ Ecological, evolutionary & environmental sciences

For a reference copy of the document with all sections, see nature.com/documents/nr-reporting-summary-flat.pdf

Behavioural & social sciences study design

All studies must disclose on these points even when the disclosure is negative.

Study description	Combination of statistical analyses and quantitative carbon footprint estimations using a prospective lifecycle assessment framwork.
Research sample	Statistical analyses are performed on vehicle retirement statistics for the Swedish passenger car fleet.
Sampling strategy	The filtered dataset includes 442,395 observations. Stratified random sampling is used to create a new dataset for analyzing the influence of increasing driving intensity since only a small share of total current vehicle retirement represents cars with high average annual driving intensity. The strata and random sample size are set to maximize the amount of information about vehicles with high driving intensity while also ensuring high enough sample size to enable further statistical analysis, see details in the Supplementary Tables 1-3.
Data collection	No primary data collection is done. Primary data are collected in accordance with Swedish Law and provided by the governmental agency Transport Analysis.
Timing	Statistics on Swedish vehicle retirement is for 2014-2018
Data exclusions	Filtering of the dataset and the stratification used are described in Methods and in detail in the Supplementary Table 4.
Non-participation	Not applicable.
Randomization	Not applicable.

Reporting for specific materials, systems and methods

We require information from authors about some types of materials, experimental systems and methods used in many studies. Here, indicate whether each material, system or method listed is relevant to your study. If you are not sure if a list item applies to your research, read the appropriate section before selecting a response.

Materials & experimental systems

n/a	Involved in the study	
☒	☐	Antibodies
☒	☐	Eukaryotic cell lines
☒	☐	Palaeontology and archaeology
☒	☐	Animals and other organisms
☒	☐	Human research participants
☒	☐	Clinical data
☒	☐	Dual use research of concern

Methods

n/a	Involved in the study	
☒	☐	ChIP-seq
☒	☐	Flow cytometry
☒	☐	MRI-based neuroimaging
